# Novel metabolic biomarkers related to sulfur-dependent detoxification pathways in autistic patients of Saudi Arabia

**DOI:** 10.1186/1471-2377-11-139

**Published:** 2011-11-04

**Authors:** Yusra A Al-Yafee, Laila Y Al- Ayadhi, Samina H Haq, Afaf K El-Ansary

**Affiliations:** 1 Biochemistry Department, Science College, King Saud University, P.O box 22452, Zip code11495, Riyadh, Saudi Arabia; 2 Autism Research and Treatment Center, Riyadh, Saudi Arabia; 3 Shaik AL-Amodi Autism Research Chair, King Saud University, Riyadh, Saudi Arabia; 4 Department of Physiology, Faculty of Medicine, King Saud University, Riyadh, Saudi Arabia

## Abstract

**Background:**

Xenobiotics are neurotoxins that dramatically alter the health of the child. In addition, an inefficient detoxification system leads to oxidative stress, gut dysbiosis, and immune dysfunction. The consensus among physicians who treat autism with a biomedical approach is that those on the spectrum are burdened with oxidative stress and immune problems. In a trial to understand the role of detoxification in the etiology of autism, selected parameters related to sulfur-dependent detoxification mechanisms in plasma of autistic children from Saudi Arabia will be investigated compared to control subjects.

**Methods:**

20 males autistic children aged 3-15 years and 20 age and gender matching healthy children as control group were included in this study. Levels of reduced glutathione (GSH), total (GSH+GSSG), glutathione status (GSH/GSSG), glutathione reductase (GR), glutathione- s-transferase (GST), thioredoxin (Trx), thioredoxin reductase (TrxR) and peroxidoxins (Prxs I and III) were determined.

**Results:**

Reduced glutathione, total glutathione, GSH/GSSG and activity levels of GST were significantly lower, GR shows non-significant differences, while, Trx, TrxR and both Prx I and III recorded a remarkably higher values in autistics compared to control subjects.

**Conclusion:**

The impaired glutathione status together with the elevated Trx and TrxR and the remarkable over expression of both Prx I and Prx III, could be used as diagnostic biomarkers of autism.

## Background

Autism spectrum disorders (ASDs) are prevalent neurodevelopmental disorders that, based on a recent survey, affect not less than 1 in 150 children born [1]. Lastly, in Saudi Arabia (population under 23 million) there were 42 500 confirmed cases of autism in 2002, and many more cases remain undiagnosed [2]. ASD diagnoses are characterized by impairments in social relatedness and communication, repetitive behaviours', abnormal movement patterns, and sensory dysfunction [3]. Further, common co-morbidity conditions often associated with an ASD diagnosis include gastrointestinal disease and dysbiosis [4], autoimmune disease [5], and mental retardation [6].

Several lines of evidence support an association of oxidative stress with ASD in at least some cases. First, there is evidence of reduced endogenous antioxidant capacity. Specifically, reduced enzymatic activities of glutathione peroxidase (GPX) [6-8], superoxide dismutase (SOD) [7,8] and catalase [9,10], and reduced levels of total glutathione (GSH), GSH/GSSG and cysteine [11] have been reported. Levels of exogenous antioxidants were also reportedly reduced in autism, including vitamin C, vitamin E and vitamin A in plasma, and zinc and selenium in erythrocytes [12]. A second indicator of altered oxidative stress in autism is derived from evidence of impaired energy metabolism [13]. Magnetic resonance spectroscopic study of the brains of autistic individuals showed reduced synthesis of ATP [14]. In addition, higher lactate [13,15,16] and pyruvate [17], levels have been reported. Third, there have been reports of improvement in certain behaviours following antioxidant administration to individuals with autism. In double-blind, placebo-controlled trials, high-dose vitamin C [18] or carnosine [19], improved autistic behaviour over baseline observations. Likewise, children with autism, who had decreased blood levels of the antioxidants GSH and cysteine as well as a decreased GSH/GSSG ratio compared with controls, had increases of these following a 3-week supplementation with betaine and folinic acid [11]. Fourth, increased excretion of oxidative stress biomarkers has been reported in children with autism. Specifically, the excretion of a F2 isoprostane, 8 isoprostaglandin F2a is increased in children with autism spectrum disorders [20]. This isoprostane is a product of nonenzymatic oxidation of arachidonic acid and is widely recognized as a reliable marker of lipid peroxidation [21]. Furthermore, nitric oxide, a free radical that can block energy production, was found to be increased in autism as compared to age and sex-matched controls [8]. In addition, elevated nitrite concentrations have been detected in individuals with autism along with elevations of thiobarbituric acid reactive substances and xanthine oxidase activity in red cells [22]. Consistent with the increased oxidative stress biomarkers, children with ASD were found to have increased body burdens of environmental toxins that may generate oxidative stress [23-26]. Taken together, these lines of evidence suggest that it is likely that at least some children with autism exhibit enhanced oxidative stress. However, none of these observations suggest how oxidative stress can lead to autism.

The formation of cross-links between the SH groups of cysteine amino acids to form disulfide bridges is an important process for maintaining the 3-dimensional structure of many proteins and enzymes. A cysteine thiol group is also the active site of some enzymes. Abnormally elevated thiols may possibly affect protein synthesis or enzyme function through disulfide bonding to the cysteinyl groups at structurally or enzymatically important sites, or by acting upon existing disulfide bridges. Elevated cysteine can interact with immunoglobulins and components of the complement pathway to reduce the clearance of immune complexes, a process which may be important in autoimmune-related diseases [27].

Such strong evidence of the contribution of oxidative stress in the aetiology of autism together with the highlighted role of the thiol redox couple in maintaining normal structure and function of many enzymatic and non-enzymatic antioxidant initiated our interest to measure glutathione and thioredoxin metabolism -related parameters in an attempt to understand how oxidative stress could lead to autism in a Saudi population.

## Methods

### Subjects and methods

The study protocol followed the ethical guidelines of the most recent Declaration of Helsinki (Edinburgh, 2000). All subjects enrolled in the study had written informed consent provided by their parents and assented to participate if developmentally able. Subjects for this study were enrolled through the ART Center (Autism Research & Treatment Center) clinic. The ART Center clinic sample population consisted of children diagnosed on the ASD. The diagnosis of ASD was confirmed in all subjects using the Autism Diagnostic Interview-Revised (ADI-R) and the Autism Diagnostic Observation Schedule (ADOS) and 3DI (Developmental, dimensional diagnostic interview). The ages of all autistic children range between 3 and 16 years old. All were males, non verbal. Intelligence quotient (IQ) for all autistic children was below 80. All were simplex cases. All are negative for fragile × gene study. The control group recruited from well baby clinic at king Khaled University Hospital and they were 3-16 year old. All participating subjects were excluded from the investigation if they had dismorphic features, tuberous sclerosis, angleman syndrome, or other serious neurological (e.g., seizures), psychiatric (e.g., bipolar disorder) or known medical conditions. All participants were screened via parental interview for current and past physical illness. Children with known endocrine, cardiovascular, pulmonary, liver, kidney or other medical disease were excluded from the study.

### Ethics approval and consent

This work was ethically approved by the ethical committee of King Khalid Hospital, King Saud University (Approval number is 11/2890/IRB). A written consent was obtained from the parents of each individual case, according to the guidelines of the ethical committee.

### Samples collection

After overnight fast, 10 ml blood samples were collected from both groups in test tubes containing heparin as anticoagulant. Centrifugation was done; plasma was obtained and deep frozen (at -80°C) until analysis time.

### Chemicals and kits

All chemicals and kits used in this study were of analytical grade, a product of Sigma (USA), Biovision, Randox, or Northwest Company.

### Biochemical assays

#### Measurement of glutathione status

Measurement of reduced GSH, total glutathione and GSH/GSSG ratio were assayed based on the glutathione recycling system by 5, 5'-Dithio-bis (2-nitrobenzoic acid) (DTNB) and glutathione reductase.

#### Determination of glutathione reductase activity (GR)

GR was measured by following the reduction in the absorbance as NADPH converted to NADP at 340 nm during the reduction of GSSG to GSH [28].

#### Determination of glutathione-S-transferase activity (GST)

The GST activity was assessed using (Biovision, USA) assay kit. Based upon that GST-catalysed reaction between GSH and the GST substrate, CDNB (1-chloro-2, 4-dinitrobenzene). The GST-catalyzed formation of GS-DNB produces a dinitrophenyl thioether which can be detected by spectrophotometer at 340 nm.

#### Determination of thioredoxin reductase activity

TR activity was measured using commercially available kit (Biovision, USA) according to the following principle:In the assay TR catalyzes the reduction of 5, 5'-dithiobis (2-nitrobenzoic) acid (DTNB) with NADPH to 5-thio-2-nitrobenzoic acid (TNB2-), which generate a strong yellow color (λmax = 412 nm). Since in crude biological samples other enzymes, such as glutathione reductase and glutathione peroxidase, can also reduce DTNB, therefore, TR specific inhibitor is utilized to determine TR specific activity. Two assays were performed: the first measurement is of the total DTNB reduction by the sample, and the second one is the DTNB reduction by the sample in the presence of the TR specific inhibitor. The difference between the two results is the DTNB reduction by TR.

#### Determination of Thioredoxin I level

The Thioredoxin 1 (Trx 1) was assessed based on a sandwich Enzyme- Linked Immunosorbent Assay (ELISA) (product of northwest company). The microtiter plate provided has been pre-coated with a monoclonal antibody specific to human Trx1. This stationary phase antibody binds sample or standard Trx 1 while nonbound proteins are removed by washing. Next, bound Trx 1 is tagged with a biotin-conjugated monoclonal antibody specific for Trx1 followed by Avidin conjugated to Horseradish Peroxidase (HRP). Subsequent addition of TMB substrate solution causes blue color (650 nm) development proportional to the amount of Trx1 originally captured by the stationary phase antibody.

#### Determination of Peroxiredoxin I and III levels

The Peroxiredoxin 1(Prx1) and III (Prx3) were assessed based on a sandwich Enzyme- Linked Immunosorbent Assay (ELISA) (product of northwest company) similar to that used in thioredoxin level assay discussed previously with one exception. The microtiter plate provided has been pre-coated with a monoclonal antibody specific to human Prx1 or Prx 3 instead of Trx 1antibody.

## Results

**Figure 1:**
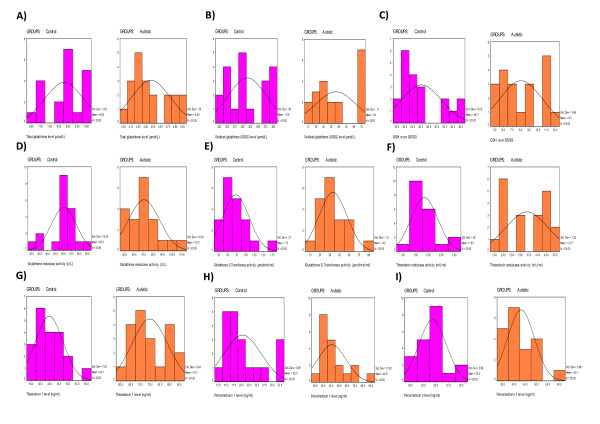
**Normal distribution of the measured parameters in plasma of control and autistic patients**.

Table 1 and figures 1A-I demonstrate the total glutathione, GSSG and GSH/GSSG in plasma of control and autistic children. It is clear from the table that these parameters recorded significantly impaired levels in autistic samples when compared to age - matching controls. Figure 1A represents the distribution of total GSH in autistic and control group. It could be easily observed that 20/20 of the autistic children have total GSH level values in (μmole/L) lower than the minimum value recorded in control group. Autistic children recorded a remarkable percentage decrease (-46.15%) compared to age-matching control. Moreover while GSSG shows remarkable higher values in autistics, recording 70% increase, GSH/GSSG ratio was significantly lower, with percentage decrease of (-70%) relative to control. Distribution of these two parameters confirmed their altered values in most of the studied autistic subjects (Figure 1B &1C).

**Table 1:** Mean ± S.D of all the measured parameters, percentage changes of autistic values relative to controls, and significant levels between both groups.

Parameter	Group	N	**Mean ± S.D**.	Percentage change	P value
**Total glutathione (μmol/L)**	Control	20	8.28 ± 1.03	100.00	0.001
		
	Autistic	20	4.46 ± 0.33	53.85	

**Oxidized glutathione (GSSG) (μmol/L)**	Control	20	0.32 ± 0.06	100.00	0.001
		
	Autistic	20	0.54 ± 0.17	170.84	

**GSH/GSSG**	Control	20	26.07 ± 5.03	100.00	0.001
		
	Autistic	20	8.03 ± 2.46	30.79	

**Glutathione reductase(U/L)**	Control	20	60.19 ± 15.42	100.00	0.052
		
	Autistic	20	70.25 ± 16.35	116.71	

**Glutathione S transferase (μmol/min/ml)**	Control	20	0.73 ± 0.37	100.00	0.002
		
	Autistic	20	0.42 ± 0.18	57.48	

**Peroxiredoxin 1 (ng/ml)**	Control	20	20.25 ± 5.99	100.00	0.001
		
	Autistic	20	40.75 ± 17.92	201.23	

**Peroxiredoxin 3 (ng/ml)**	Control	20	24.30 ± 2.69	100.00	0.001
		
	Autistic	20	43.05 ± 5.86	177.16	

**Thioredoxin 1 (ng/ml)**	Control	20	44.71 ± 7.43	100.00	0.001
		
	Autistic	20	74.70 ± 9.04	167.09	

**Thioredoxin reductase (mU/ml)**	Control	20	1.83 ± 0.52	100.00	0.001
		
	Autistic	20	3.37 ± 1.22	184.15	

Independent student's t-test between the control and autistic groups and percentage change in total glutathione level, oxidised glutathione (GSSG), GSH/GSSG, glutathione reductase, glutathione -S- transferase, peroxiredoxin 1, peroxiredoxin 3 level, thioredoxin 1, thioredoxin reductase (mU/ml).

The significant reduction in plasma glutathione s transferase activity in autistic patients compared to control (-42.52%) is shown in the graphical distribution demonstrated in Figure 1E. 10/20 of control subject recorded value of 0.75 (μmol/min/ml) or higher compared to 19/20 of autistic subjects that recorded lower values.

Table 1 together with Figure 1F show an obvious significant elevation on thioredoxin reductase activity in autistic patients (84.15%) compared to age and sex matching control. Results shows that 20/20 of control subject record enzymatic activity equal to 3 (mU/ml) or lower compared to only half of the autistic participants recorded this values. Moreover, results represented in table 1 and Figure 1G demonstrate the levels of thioredoxin in plasma of control and autistic children. It could be easily noticed from the table that this parameter recorded significantly raised levels in autistic samples when compared to age - matching controls. Graphical distribution of Trx in autistic and control groups show that 20/20 of the autistic children have Trx levels range between 59.93-89.7 ng/ml while only 1/20 of the control samples recorded values within this range. Autistic children recorded a high percentage increase (67.09%) compared to age-matching control.

Tables 1 and Figures 1H &I demonstrate the peroxiredoxin I and III levels in control and autistic subjects. It could be easily noticed that prx I and prx III levels are significantly higher in autistic patients compared to control. Prx I shows almost 3 folds higher values with high percentage increase (101.23%) compared to control. In case of prx III levels the highest value recorded in control subjects was less than the lowest value recorded in autistic patients with no observed overlapping between the two groups.

**Figure 2:**
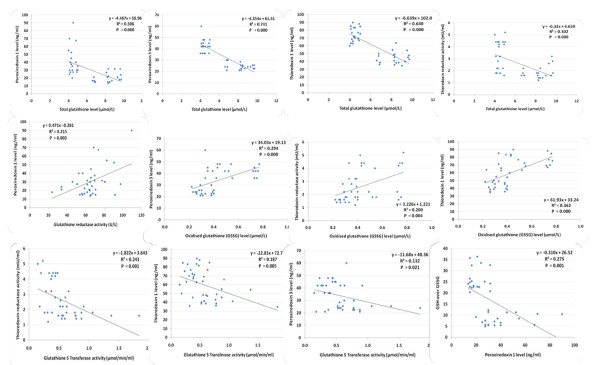
**Pearson's correlations of the most significant positive and negative correlated variables listed in table 3**.

**Figure 3:**
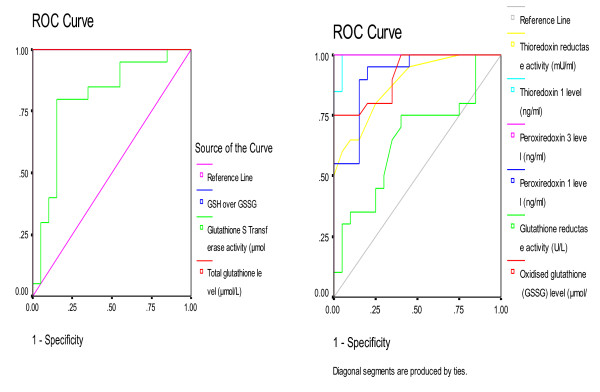
**ROC Curve of the measured parameters showing, area under the curve (AUC), specificity and sensitivity**.

Table 2 and Figure 2 demonstrate Pearson correlations between all the measured parameters. It could be easily noticed that most of the measured parameters are either positively or negatively correlated with different levels of significance. Only those which demonstrate the most significant correlations between glutathione in one hand and thioredoxin- related parameters in the other hand are presented in Figure 3.

**Table 2:** Pearson correlation test showing correlation significance differences between the different measured parameters:

Parameters	Pearson Correlation	**Sig**.	
**Total glutathione ~GSSG**	-0.536	0.000	N^b^

**Total glutathione level ~ Glutathione reductase**	-0.339	0.032	N^b^

**Total glutathione ~Glutathione S Transferase**	0.502	0.001	P^a^

**Total glutathione ~ Peroxiredoxin 1**	-0.553	0.000	N^b^

**Total glutathione ~ peroxiredoxin 3**	-0.861	0.000	N^b^

**Total glutathione ~ thioredoxin 1**	-0.800	0.000	N^b^

**Total glutathione ~thioredoxin reductase**	-0.550	0.000	N^b^

**Total glutathione ~ GSH/GSSG**	0.871	0.000	P^a^

**GSSG ~ glutathione S Transferase**	-0.436	0.005	N^b^

**GSSG ~ peroxiredoxin 3**	0.543	0.000	P^a^

**GSSG ~ thioredoxin 1**	0.602	0.000	P^a^

**GSSG ~ thioredoxin reductase**	0.447	0.004	P^a^

**GSSG ~ GSH/GSSG**	-0.801	0.000	N^b^

**Glutathione reductase) ~ peroxiredoxin 1**	0.464	0.003	P^a^

**Glutathione S transferase ~ peroxiredoxin 3**	-0.364	0.021	N^b^

**Glutathione S Transferase ~ thioredoxin 1**	-0.434	0.005	N^b^

**Glutathione S Transferase ~ thioredoxin reductase**	-0.494	0.001	N^b^

**Glutathione S Transferase ~ GSH/GSSG**	0.513	0.001	P^a^

**Peroxiredoxin 1 ~ Peroxiredoxin 3**	0.519	0.001	P^a^

**Peroxiredoxin 1 ~ thioredoxin 1**	0.517	0.001	P^a^

**Peroxiredoxin 1 ~ thioredoxin reductase**	0.446	0.004	P^a^

**Peroxiredoxin 1 ~ GSH/GSSG**	-0.525	0.001	N^b^

**Peroxiredoxin 3 ~ thioredoxin 1**	0.873	0.000	P^a^

**Peroxiredoxin 3 ~ thioredoxin reductase**	0.617	0.000	P^a^

**Peroxiredoxin 3 ~ GSH/GSSG**	-0.803	0.000	N^b^

**Thioredoxin 1 ~ thioredoxin reductase**	0.615	0.000	P^a^

**Thioredoxin 1 ~ GSH/GSSG**	-0.779	0.000	N^b^

**Thioredoxin reductase ~ GSH/GSSG**	-0.558	0.000	N^b^

**^a ^**Positive Correlation.

**^b ^**Negative Correlation.

Table 3 and Figure 3 demonstrate the receiver operating characteristics of the measured parameters. Area under the curve, specificity and sensitivity of the measured parameters are illustrated

**Table 3:** ROC analysis of the measured parameters showing area under the curve,cutoff values, sensitivity and specificity.

Parameter	Area under the curve	Cutoff value	Sensitivity %	Specificity %
**Total glutathione**	1.000	9.320	100.0%	20.0%

**Oxidised glutathione (GSSG)**	0.919	0.380	80.0%	80.0%

**GSH/GSSG**	1.000	31.100	100.0%	20.0%

**Glutathione reductase**	0.650	75.620	30.0%	90.0%

**Glutathione- S- transferase**	0.803	1.100	100.0%	15.0%

**Peroxiredoxin 1**	0.915	26.240	90.0%	80.0%

**Peroxiredoxin 3**	1.000	26.990	100.0%	85.0%

**Thioredoxin 1**	0.993	52.140	100.0%	85.0%

**Thioredoxin reductase**	0.881	2.350	65.0%	85.0%

## Discussion

Clinical and preclinical investigations of the actions of antioxidative defence systems in the brain suggest several ways in which ongoing oxidative stress as a primary phenomenon might impact the occurrence and severity of autism [11]. Mitochondrial dysfunction, leaky blood brain barrier, higher levels of circulating cytokines and autoimmune response to brain antigen may be promoted by oxidative generation of neoepitopes, which occurs via oxidative alteration of neurodevelopmental proteins [29-31].

All cellular functions are affected by the prevailing redox status, and sulfur metabolism plays a central role in maintaining a redox potential that is favorable for homeostasis. In the present study, the significantly decreased plasma total GSH levels (table 1 and Figure 1A) among the participants diagnosed with autism is of concern. GSH is a tripeptide of cysteine, glycine, and glutamate that is synthesized in every cell of the body. The essential intracellular reducing environment is maintained by the high ratio of reduced GSH/GSSG [32]. The GSH redox equilibrium regulates a wide range of functions that include nitrogen and oxygen free radical scavenger [33], protein redox status and enzyme activity [34], cell membrane integrity and signal transduction [35,36], transcription factor binding and gene expression [37], phase II detoxification [38], and apoptosis [39]. The observed depletion of total GSH could be supported through considering the recent reports which also demonstrate that, compared to controls, patients with autism showed reduced levels of plasma total glutathione [40,41]. Oxidative stress caused by GSH depletion may also play a role in the increased male to female ratio observed in autism. Because of lower levels of reduced glutathione, mitochondria from males, when compared to females, are more vulnerable to oxidative stress [42]. In animal studies, due to lower enzyme activity levels of superoxide dismutase (SOD) and glutathione peroxidase in males, oxidative damage to mitochondrial DNA is 4-fold higher in males compared to females [43]. The obtained higher GSSG levels as a reliable marker of intracellular oxidative stress in autistics (table 1 and Figure 1B) could find a support in the previous work of James et al., 2004 [12] who recorded 72% higher GSSG in children with autism when compared to neurotypical children.

The unexpected, non-significant change in the activity of glutathione reductase in autistic children when compared to normal controls (table 1 and Figure 1D) could be explained on the basis that under physiological conditions, glutathione reductase activity is sufficient to maintain an elevated reduced/oxidized glutathione ratio. However, excessive intracellular oxidative stress, exceeding the capacity of glutathione reductase will induce export of GSSG to the plasma in an attempt to regain intracellular redox homeostasis [44].

The significant reduction in plasma glutathione -S- transferase in plasma of autistic patients compared to control subjects (table 1 and Figure 1E) could be easily correlated to lack of substrate availability in autistic subjects i.e. reduced glutathione (GSH) that was previously observed in autistic patients [11,40,41]. The recorded reduction in this essential detoxifying enzyme could explain the observed poor detoxification power in autistic patients, such as in case of mercury or lead toxicity [25,26,44]. The lower activity levels of glutathione-S- transferase, reported in the present study is in good agreement with the previous study done by Hung et al [45] in which they proved that Glutathione-*S*-transferase activity was reduced in autistic patients compared to control. Moreover it could find a support in a genetic study on this enzyme which revealed that M1 (GSTM1), is reduced or absent in individuals carrying the GSTM1*0 (null) allele, increasing their sensitivity to xenobiotics [46] In addition, two recent studies have reported an association between the null allele and autism suggesting that GST contributes to the risk of oxidative stress and autism [46,47].

**Figure 4:**
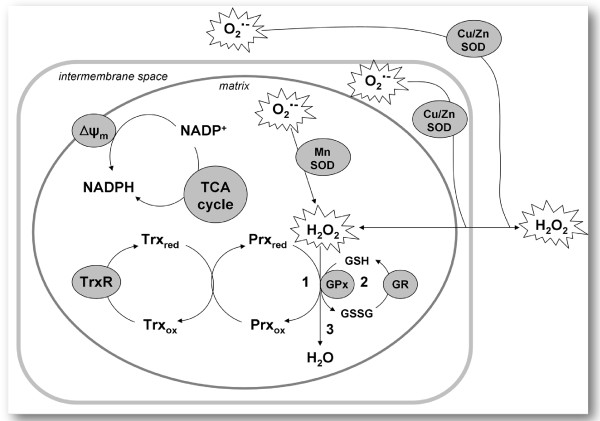
**Proposed model of H2O2 removal in brain mitochondria**[51] .

The key biological activities of Trx that are applicable to human disease can be categorized as antioxidant, growth promoting, anti-apoptotic and inflammation modulating [48]. Beyond its protective role, the Txn system is involved in various cellular processes, such as cell-cell communication, transcriptional regulation, cell signalling, and DNA synthesis [49]. Any particular biological property of thioredoxin (Trx) is unlikely to be either 'good' or 'bad' in human diseases [50]. Over expression of TrxR as a major antioxidant enzyme, recorded in the present study could support the hypothesis stated that oxidative stress is linked with the etiology of autism [11,47]. One of the most important roles of the Trx/TrxR system is to maintain reduced redox status and prevent oxidative stress. This function is holding up mainly by the peroxiredoxin system. Recently Drechsel and Patel [51] demonstrate that Trx/Prx is the major contributing enzyme system to respiration-dependent H_2_O_2 _removal in brain mitochondria, while GSH/GPx and non-enzymatic systems show only minor contributions. This pathway could be easily followed in Figure 4.

For all of that, in this study we also measured different Prx levels to obtain their assumed role in autism. The demonstrated over expression of both Prx I and Prx III as biomarkers of oxidative stress in autistic patients compared to age and gender-matching control subjects (table 1 and Figure 1H &1I), could support their roles in the pathology of autism. Besides their cytoprotective antioxidant function, Prxs appeared to play an important role in cell proliferation, differentiation, immune response, protection of oxidant-sensitive proteins, regulation of cellular H_2_O_2 _and control of apoptosis, processes involving a redox signalling [52]. Prx3 overexpression alters the mitochondrial membrane potential, reduces endogenous cellular H_2_O_2 _levels. The results of Nonn et al [53] suggested that mitochondrial Prx3 is an important regulator of H_2_O_2 _in the cell. They reported that, at low physiological levels of H_2_O_2_, Prx3 inhibits the growth-stimulating effects of H_2_O_2_, while at higher levels of H_2_O_2 _generation; Prx3 protects cells against apoptosis [54].

Based on this information, the recorded raised levels of Trx, TrxR and Prxs of the present study could be related to the previous work of Al-Gadani et al [11] in which they proved that Saudi autistic children are under H_2_O_2 _stress due to over expression of SOD and a slightly lower activity of catalase.

The lack of satisfactory specificity values in glutathione-related parameters could be supported through considering the facts that glutathione system is known to be altered in many chronic neurological disorders [11,54-56].

## Conclusion

The satisfactory high values of both sensitivity and specificity recorded by Trx, TrxR and Prxs could help to suggest them as biomarkers for the diagnosis of autism in Saudi population. Based on this study which confirmed the impaired antioxidant status in Saudi autistics, early intervention through supplementation of perfect and safe antioxidants as omega-3, carnosine, selenium and others could be helpful because no doubt that autistic children who undergo intensive intervention, be it behavioural or developmental, do better than children who don't.

## Abbreviations

ART: Autism Research and Treatment centre; ASD: Autism Spectrum Disorder; IQ: Intelligence quotient; GSH: reduced glutathione; GSH+GSSG: total glutathione; GSH/GSSG: glutathione status; GR: glutathione reductase; GST: glutathione- s-transferase; Trx: thioredoxin; TrxR: thioredoxin reductase (TrxR); Prxs I and III: peroxidoxins I and III; H_2_O_2_: hydrogen peroxide; DNA: deoxyribonucleic acid.

## Competing interests

The authors declare that they have no competing interests.

## Authors' contributions

**YA: **Carried out the biochemical assays, **LA: **Confirmed the diagnosis, provided the samples and ethical approval, **SH: **Participated in performing the statistical analysis, **AE: **Designed the study and drafted the manuscript. All authors have read and approved the final manuscript.

## Pre-publication history

The pre-publication history for this paper can be accessed here:

http://www.biomedcentral.com/1471-2377/11/139/prepub
